# Breads Fortified with Freeze-Dried Vegetables: Quality and Nutritional Attributes. Part 1: Breads Containing Oil as an Ingredient

**DOI:** 10.3390/foods5010019

**Published:** 2016-03-14

**Authors:** Viren Ranawana, Vassilios Raikos, Fiona Campbell, Charles Bestwick, Phyllis Nicol, Lesley Milne, Garry Duthie

**Affiliations:** Natural Products Group, Rowett Institute of Nutrition and Health, University of Aberdeen, Aberdeen AB25 2ZD, UK; v.raikos@abdn.ac.uk (V.R.); fiona.campbell@abdn.ac.uk (F.C.); C.Bestwick@abdn.ac.uk (C.B.); p.nicol@abdn.ac.uk (P.N.); l.milne@abdn.ac.uk (L.M.); g.duthie@abdn.ac.uk (G.D.)

**Keywords:** bread, vegetables, oxidative stability, oil, storage properties, digestion

## Abstract

There is increasing emphasis on reformulating processed foods to make them healthier. This study for the first time comprehensively investigated the effects of fortifying bread (containing oil as an ingredient) with freeze-dried vegetables on its nutritional and physico-chemical attributes. Breads fortified with carrot, tomato, beetroot or broccoli were assessed for nutrition, antioxidant potential, storage life, shelf stability, textural changes and macronutrient oxidation. Furthermore, using an *in vitro* model the study for the first time examined the impact of vegetable addition on the oxidative stability of macronutrients during human gastro-intestinal digestion. As expected, adding vegetables improved the nutritional and antioxidant properties of bread. Beetroot and broccoli significantly improved bread storage life. None of the vegetables significantly affected bread textural changes during storage compared to the control. Lipid oxidation in fresh bread was significantly reduced by all four types of vegetables whilst protein oxidation was lowered by beetroot, carrot and broccoli. The vegetables demonstrated varying effects on macronutrient oxidation during gastro-intestinal digestion. Beetroot consistently showed positive effects suggesting its addition to bread could be particularly beneficial.

## 1. Introduction

There is presently much emphasis on reformulating processed foods to make them healthier. Increasing consumer demands for healthier foods has resulted in a drive towards producing “clean label” products that are nutritionally superior and do not contain synthetic additives. Macronutrient oxidation is a particular concern for the food industry as it adversely affects organoleptic properties and shelf life, and next to microbes is the second greatest contributor to food spoilage [1]. Most processed foods therefore have synthetic additives to curtail oxidation, despite an increasing consumer pressure to reduce their use.

The oxidation of lipids and proteins in foods has implications both from consumer health and Industrial perspectives. The oxidation of these macronutrients leads to the development of toxic end-products that can contribute to disease pathogenesis by affecting the stability and biochemistry of cells and genes. For example, aldehydes such as malondialdehyde which results from the oxidation of polyunsaturated fatty acids exert mutagenic and artherogenic effects, while protein oxidation products such as carbonyls are implicated in cell ageing and age-related diseases [2,3]. The alimentary tract of humans can often be oxygen-rich and provide an ideal environment for the oxidation of dietary proteins and fats [4]. Thus it is imperative that dietary macronutrients are protected from oxidation to facilitate their optimal digestion.

Plant products are rich in natural antioxidants and functional components which have been suggested to curtail oxidation of foods [1]. In agreement, work from our group showed that adding vegetables to burgers and emulsions improved their oxidative stability and shelf life [5,6,7]. However, as antioxidants in vegetables are sensitive to food processing conditions their efficacy in processed food systems needs to be evaluated product-specifically.

The present study investigated the oxidative stability of breads incorporated with vegetables. Bread is the most widely consumed staple in many parts of the world including Europe, Asia and the Americas. It inherently has a short shelf life of no longer than 3 days, and thus commercially produced breads often contain additives to extend shelf life [8]. Vegetables containing natural antioxidants may be an effective natural substitute for prolonging the shelf life of breads. Incorporating vegetables to breads may also serve as an alternative route by which this important food group can be introduced into a population’s diet. Despite dietary recommendations to consume at least 400 g of fruits and vegetables per day many Europeans fail to meet this target [9]. As the most popular staple bread could be used to increase fruit and vegetable intakes, similar to how it has been previously used to deliver nutrients and functional foods [10,11].

Thus the addition of vegetables to breads may have both Industrial and public health benefits. Although previous work has looked at the feasibility of vegetable addition into bread [12] no studies have comprehensively assessed its functional and nutritional effects. Furthermore, there is no data on how vegetable addition affects macronutrient oxidation in processed foods during gastro-intestinal digestion. The prevalent high consumption of protein and fat-rich foods [13] and the pro-oxidant environment of the human alimentary tract underscore the importance of devising ways of protecting these macronutrients from oxidation during digestion. To our knowledge this is the first study to comprehensively characterise the nutritional and oxidative-stability attributes of breads supplemented with vegetables. The specific objective of this study was to assess the nutritional, storage and antioxidant implications of adding vegetables to bread (containing fat as an ingredient), and its effects on oxidative stability during storage and gastrointestinal digestion. The study hypothesised that adding vegetables will improve nutritional and functional attributes of bread, and oxidative stability during storage and gastrointestinal digestion.

## 2. Materials and Methods

### 2.1. Test Breads

The test breads were made at the Rowett Institute of Nutrition and Health (RINH). Five bread types were prepared (plain (control), carrot, tomato, broccoli, and beetroot). The ingredients used for the plain bread were strong white wheat flour (59% *w*/*w*; Allinson flour, Peterborough, UK), water (30%), vitamin E stripped corn oil (7.1%; MP Biomedicals, California, CA, USA), yeast (1.8%; Tesco Stores Ltd., Dundee, UK) and salt (1.2%). The vegetable breads contained the same ingredients with the exception that 10% of the flour (by weight) was substituted with freeze dried vegetable powder. All the breads were prepared using the same following methodology: the yeast was dissolved in the water (pre-warmed to 40 °C), added to the dry ingredients and mixed to form doughs. The doughs were kneaded for 10 min and subjected to a first prove for 25 min at room temperature (until doubled in size). They were then knocked back, made up, placed in bread tins (21 × 11 × 6 cm), proved for 40 min and baked at 200 °C for 30 min. The cooled breads were sliced into 1.5 cm thick slices and half of the loaf was used for texture analyses. The other half was freeze dried (Model HS1, Frozen in Time Ltd., York, UK), ground to a fine powder (model ZX809X, Wahl, Kent, UK) stored in black polyethylene bags at −70 °C, and used for all other analyses.

The freeze dried vegetable powders were prepared at RINH. Fresh carrot, tomato, broccoli and beetroot were purchased from local supermarkets. These were washed, chopped, freeze dried (Model HS1, Frozen in Time Ltd., York, UK), ground to a fine powder (Blixer 2, Robot Coupe, Isleworth, UK) and stored in black polyethylene bags at 6 °C until used for making the breads. The broccoli was cut into florets prior to drying. The tomato was dried with the seeds, and the carrot and beetroot were dried with their peel intact.

Routine analytical procedures were followed to determine the proximate composition of the breads (Table 1). In addition, several micronutrients with recognised antioxidant activity (vitamin E: α and γ-Tocopherol, and carotenoids: α-Carotene, β-Carotene, β-Cryptoxanthin, Lutein, Lycopene) were quantified using high performance liquid chromatography.

### 2.2. Chemicals

Alpha amylase from porcine pancreas (U/mg), mucin, (m2378), Pepsin from porcine gastric mucosa (U/mg), Pancreatin from porcine pancreas (U/mg), Adenosine di phosphate (ADP), 1, 1, 3, 3- Tetramethoxypropan Ascorbic acid, FeSO_4_, NaCl, Disodiumorthophosphate (Na_2_HPO_4_), Pottasium dihydrogenphosphate (KH_2_PO_4_), HCl, Trichloroacetic acid (TCA), Thiobarbituric acid (TBA), 2,4-Dinitrophenylhydrazine (DNPH) phosphoric acid and Acetic acid were purchased from Sigma-Aldrich Company. Reagents used for HPLC analysis were HPLC grade and purchased from VWR International (Lutterworth, UK). FRAP reagents included sodium acetate, glacial acetic acid, 2,4,6-Tris(2-pyridyl)-*s*-triazine (TPTZ), HCl (Fisher Scientific UK Ltd), FeCl_3_·6H_2_O and FeSO_4_·7H_2_O. HORAC reagents included gallic acid, fluorescein sodium salt, H_2_O_2_, cobalt (II) fluoride and picolinic acid. All chemicals for the FRAP and HORAC assays were obtained from Sigma-Aldrich Company unless otherwise stated.

### 2.3. Antioxidant Capacity of Vegetable Breads

Aqueous extracts of the bread samples were made by homogenising 0.4 g of freeze dried bread with 9.6 mL of 0.9% NaCl. An isotonic extraction solution was used as it was a physiologically more relevant medium. The homogenates were placed on a rolling shaker for 120 min and centrifuged at 3500 rpm (CR 312, Jouan, Thermo Fisher Scientific, Renfrew, UK) for 60 min at 4 °C. The resulting supernatants were used for assaying the antioxidant capacity of the breads using the Ferric Reducing Antioxidant Potential (FRAP), and Hydroxyl Radical Averting Capacity (HORAC) methods. The method of Benzie and Strain [14] was used for assaying FRAP which is based on the reduction of colourless ferric complex (Fe^3+^ tripyridyltriazine) to the blue-coloured ferrous complex (Fe^2+^ tripyridyltriazine) by the action of electron donating antioxidants at low pH. The HORAC assay is based on the oxidation of a fluorescent probe by hydroxyl radicals via a hydrogen atom transfer process and was assayed using a kit (Product number TA30, Oxford Biomedical Research Inc., Michigan, MI, USA) [15].

### 2.4. Oxidative Stability of Breads

The effects of the vegetable powders on the oxidative stability of bread were determined with a 743 Rancimat (Metrohm, Herisau, Switzerland). Freeze-dried bread samples (3 g) were measured into reaction tubes (in duplicate) and subjected to an accelerated oxidation at 120 °C and a gas (ambient air) flow rate of 7 L/hour. The generation of oxidation products was measured and using this data induction times were interpolated as a directly proportional indicator of oxidative stability and shelf life. Analysis of all samples was carried out in independent duplicate runs.

### 2.5. Oxidation End-Products in Vegetable Breads

Lipid oxidation end-products in fresh bread were measured as Thiobarbituric acid reactive substances (TBARS) and protein carbonyls (PC). Freeze dried bread samples (~50 μg) were accurately weighed and added to 4 mL of distilled water and homogenised (Ultra-Turrax T18, Janke & Kunkel, IKA instruments, Germany) at 12,000 rpm for 10 s. The homogenates were analysed for TBARS as previously described [16]. Briefly, 1 mL of TBA reagent (0.67% TBA solution mixed in an equal volume of glacial acetic acid) was added into each tube and vortexed. The tubes were subsequently heated at 95 °C for 30 min, cooled to room temperature and centrifuged for 10 min at 3500 rpm (CR 312, Jouan, Thermo Fisher Scientific). The supernatants were analysed for TBARS content by reverse-phase HPLC using a Waters 2695 separations module coupled with a Waters 2475 fluorescence detector (Waters Ltd, Elstree, UK) and a 100 × 4.6 Phenomenex Luna 5 µ C18(2) 100 A column set at 30 °C (Phenomenex, Cheshire, UK). Separations were carried out on 20 µL samples at a flow rate of 0.8 mL/min over 20 min using a mobile phase made of 60% 50 mM KH_2_PO_4_ (pH 7.0) and 40% Methanol. Standard solutions of 1,1,3,3 Tetramethoxypropane were simultaneously analysed in triplicate for constructing calibration curves and quantification of TBARS. TBARS content was expressed as nmol per g of sample. Protein carbonyls were measured as previously described [5]. Following incubation (25 °C; 10 min) of the samples (0.1 g) with KCl (0.15 M; 1 mL) containing FeSO_4_ (1 mM) and H_2_O_2_ (1 mM) 20% TCA was added to precipitate the protein. Following the addition of 0.2% DNPH the samples were heated at 45 °C for 1 h and centrifuged at 13,000× *g* for 5 min. The supernatant was removed and the pellet was washed in ethanol:ethyl acetate (1:1 *v*/*v*) three times, dissolved in 300 µL 6 M guanidine hydrochloride and absorbance read at 370 nm. Protein content was determined using the Pierce 660 protein assay (Thermo Scientific, Paisley, UK) according to the manufacturers recommended protocol Carbonyl content was quantified using the molar extinction coefficient of 22,000 M^−1^·cm^−1^ and the results expressed as nmol of carbonyl per mg of protein.

### 2.6. Changes in Texture and Oxidative Stability during Storage

Freshly prepared breads were stored for 1, 2 and 4 days, and changes in texture were measured. Freshly baked and cooled loaves were sliced into 1.5 cm slices on the day of baking, and pairs of randomly selected slices were placed in polyethylene bags. One set of samples was analysed on the day of baking for baseline measurements (day 0). The remainder were stored for 1, 2 and 4 days in the dark at ambient temperature (21 °C). Texture profile analyses of the bread crumb was carried out at 0, 1, 2 and 4 days of storage using a texture analyser ( CT3, Brookefield Viscometers Ltd, Harlow, UK) equipped with a cylinder probe (TA11/1000, D = 25.4 mm). Each bread slice was 40% compressed twice to give a two bite texture profile. Two slices were tested for each treatment at each time point which resulted in four readings per treatment per day. Trigger load and test speed were 10 g and 0.5 mm/s respectively. The parameters hardness, cohesiveness, and gumminess were evaluated using the *in situ* software (TexturePro CT, Brookefield Viscometers Ltd, Harlow, UK). Duplicate measures were made for each bread type on each day.

### 2.7. Oxidative Stability during in Vitro Gastrointestinal Digestion

*In vitro* gastro-intestinal digestions were carried out using a system that mimics the oral, gastric and intestinal phases of human digestion and is typical of those used previously [17,18]. The compositions of the artificial juices used were (per 1000 mL); saliva (pH 6.8 ± 0.1): Na_2_HPO_4_ 2.38 g, KH_2_PO_4_ 0.19 g, NaCl 8 g, Mucin 100 mg, α- Amylase 200 U/mL; gastric fluid (2.0 ± 0.1): NaCl 2 g, Pepsin 3.2 g; Intestinal fluid (pH 8.0 ± 0.1): KH_2_PO_4_ 6.8 g, Pancreatin 10 g. Simulated saliva was prepared as described by Gawlik-Dziki [17], and simulated gastric (SGF) and intestinal (SIF) fluids according to the US Pharmacopeia [19]. The digestions were carried out in black centrifuge tubes (LightSafe, Sigma-Aldrich) placed in a shaking water bath (SB-16, Techne, Bibby Scientific Ltd, Stone, UK) adjusted to 37 °C. A sample: saliva ratio of 1:3 was maintained at the start of the digestions similar to previous studies digesting bread in *in vitro* gastrointestinal incubation systems [20].

Briefly, bread samples were weighed into 15 mL black centrifuge tubes and 3 mL of cold simulated saliva was added. The digestion tubes were then placed in the shaking water bath for 5 min to complete the oral phase of digestion (pH 6.8) and the phase halted by the addition of 0.5 mL of 0.3 M HCl. The gastric phase of digestion was initiated by the addition of 8 mL of double-strength SGF (pH 2.0) containing 0.68 mg of Ascorbic acid, 0.11 mg of FeSO_4_ and 6.8 mg of ADP. Lipid peroxidation is induced by Fe/ADP complex in the presence of Ascorbic acid and was thus added to create a pro-oxidant environment. The gastric digestion phase was continued for four hours. At the end of two hours of gastric digestion 1.5 mL volumes of gastric digesta were transferred into separate tubes containing 1.5 mL of double strength SIF (pH 8.0) and the intestinal digestion phase was carried out in parallel for two hours. At baseline, and during each of the digestion phases 0.5 mL digesta aliquots were aspirated and transferred into, (1) glass tubes containing 1 mL 20% Trichloroacetic acid for measuring concentrations of TBARS and; (2) glass tubes containing 50 µL of 0.2% Dinitrophenylhydrazine (DNPH, in 3.5 M HCl) for measuring PCs. Digesta samples collected for TBARS quantification were analysed immediately following the digestions whilst those collected for PC analysis were stored at −70 °C and analysed within 7 days. All of the bread samples were subjected to *in vitro* digestions in three independent runs and the data was pooled for analysis.

For measuring TBARS the total volume of the TCA tubes containing digesta samples were brought to 4 mL with double-distilled water, analysed using the method of Yagi (1987) [16] as previously described for the fresh bread samples and expressed as nmol per g of bread. The PC content was quantified using the above described method for fresh bread samples and expressed as pmol of protein carbonyls per g of bread.

### 2.8. Statistical Analyses

Total TBARS and protein carbonyls formed during four hours of *in vitro* gastric digestion and two hours of intestinal digestion were quantified by calculating the Areas Under the digestion Curves (AUC) using the trapezoidal rule. Data on the TBARS and protein carbonyl contents in the fresh bread samples, AUCs from gastrointestinal digestions, and the antioxidant capacity, texture analysis and Rancimat data were analysed using one-way ANOVA. Post hoc tests were carried out where significant differences were observed using the Bonferroni correction, Ryan, Einot, Gabriel and Welsch Q procedure, and Scheffe test as appropriate. A *p* < 0.05 was considered significant.

## 3. Results

### 3.1. Antioxidant Capacity of Vegetable Breads

**Figure 1:**
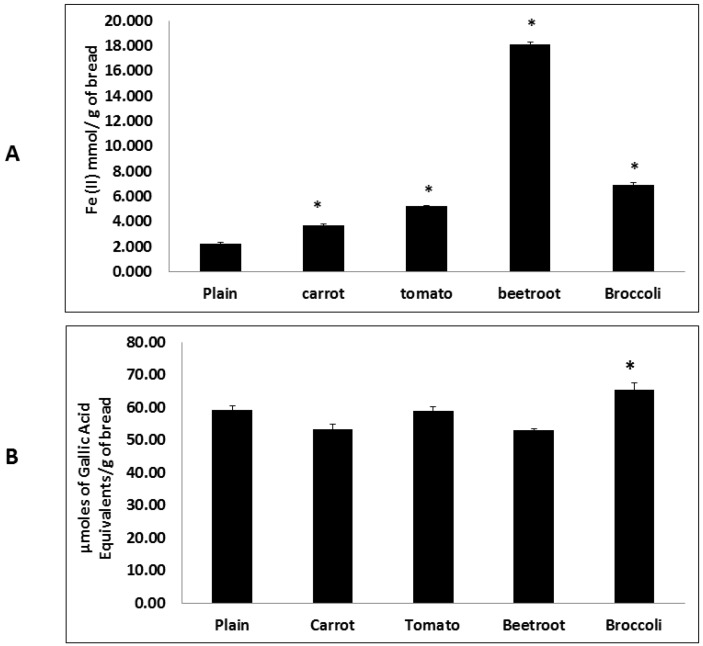
Antioxidant potential of the breads. Antioxidant potential of breads measured using FRAP (**A**) and HORAC (**B**) assays. Treatments with asterisks are significantly different to the plain bread (*p* < 0.05). Values are means ± SE (*n* = 3).

Adding vegetables significantly affected the FRAP assay associated antioxidant capacity of the vegetable breads (F(4,10) = 2042.52; *p* < 0.001) (Figure 1A). Post hoc analyses showed that all the vegetable breads had significantly higher antioxidant capacities compared to the plain bread and were also significantly different to each other. Beetroot bread demonstrated the greatest antioxidant potential and was notably higher than the others.

The HORAC assay also showed significant differences in the antioxidant potential of the breads (F(4,10) = 11.60; *p* = 0.001) (Figure 1B). However, compared to the plain bread only broccoli bread showed a significantly different (higher) antioxidant potential. The antioxidant potential of broccoli bread was also significantly higher (65.3 mM Gallic Acid Equivalents/g of sample) than in carrot and beetroot breads (53.2 and 53.1 mM GAE/g of sample respectively).

### 3.2. The Oxidative Stability of Breads

**Figure 2:**
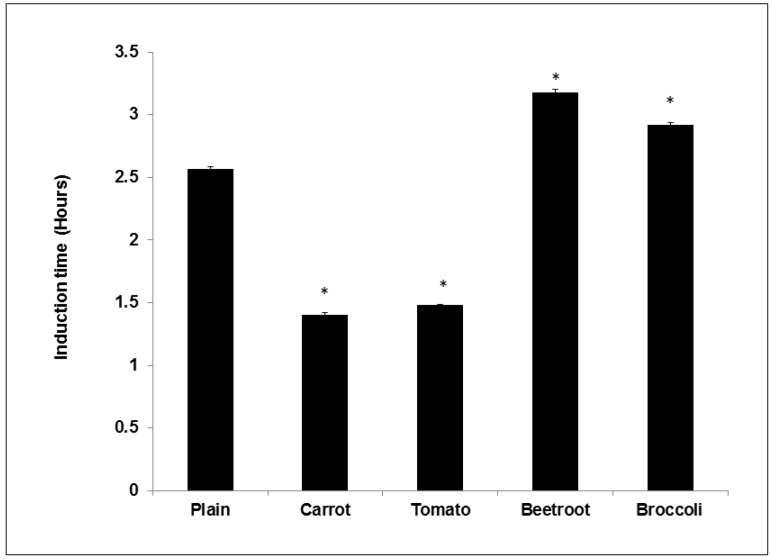
Oxidative stability of the breads measured using the Rancimat. Treatments with asterisks are significantly different to the plain bread (*p* < 0.05). Values are means ± SE (*n* = 4).

The oxidative stability of the vegetable breads (measured as the induction time using the Rancimat) were significantly different to that of the plain bread (F(4,15) = 1492.7; *p* < 0.05) (Figure 2). The beetroot and broccoli breads showed significantly longer induction times (3.2 ± 0.04 and 2.9 ± 0.04 h) compared to the control (2.6 ± 0.03 h respectively). The carrot and tomato breads demonstrated significantly shorter induction times (1.4 ± 0.05 and 1.5 ± 0.01 h respectively) compared to the plain. Correlational analysis showed that there was no association between induction time and PC contents in the samples (*r* = −0.604, *p* > 0.05). Similarly, there was no association between induction time and TBARS content in the breads (*r* = −0.545, *p* > 0.05).

### 3.3. Oxidation End-Products in Vegetable Breads

**Figure 3:**
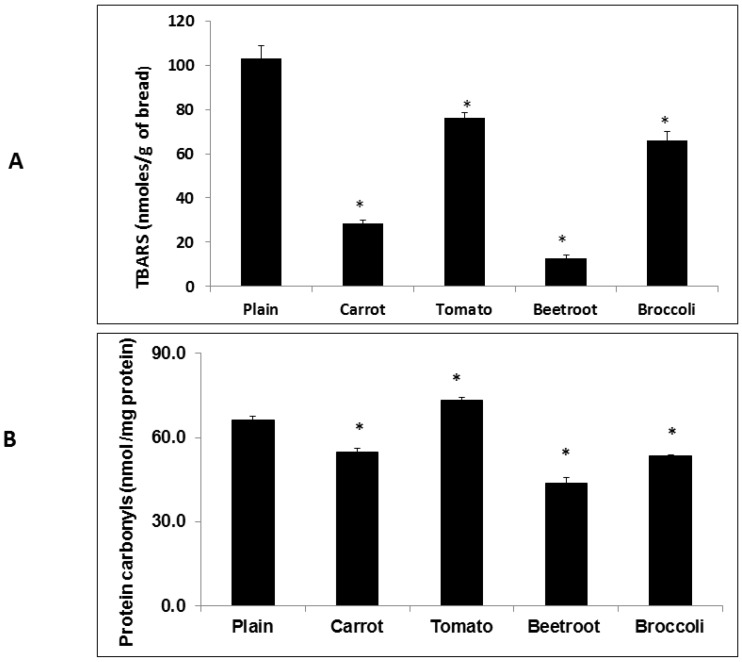
Thiobarbituric acid reactive substances (TBARS) (**A**) and Protein Carbonyl (PC) (**B**) contents in the fresh breads. Treatments with asterisks are significantly different to the plain bread (*p* < 0.05). Values are means ± SE (*n* = 3).

All four vegetable breads showed significantly lower TBARS contents compared to the plain bread (F(4,15) = 80.1; *p* < 0.05) (Figure 3A). Beetroot bread showed the lowest TBARS content (12.9 ± 3 nmol/g) compared to the control (102.9 ± 12 nmol/g), followed by carrot bread (28.4 ± 3 nmol/g). The amount of TBARS in the tomato and broccoli breads were statistically similar (76.3 ± 4 and 65.9 ± 8 nmol/g respectively). The PC content in the fresh breads also differed significantly compared to the plain bread (F(9,20) = 124.5; *p* < 0.001) (Figure 3B). Compared to the plain bread (66.3 ± 2 nmol of carbonyls/mg of protein) beetroot bread contained the lowest amount of PC (43.6 ± 4) followed by broccoli and carrot breads (53.5 ± 1 and 55.0 ± 2 respectively). Tomato bread contained a significantly higher PC content (73.3 ± 1 nmol of carbonyls/mg of protein) compared to the plain bread. Correlational analysis showed that there was a non-significant but noteworthy correlation between TBARS and PC contents in the breads (*r* = 0.78, *p* = 0.06).

### 3.4. Changes in Texture during Storage

**Figure 4:**
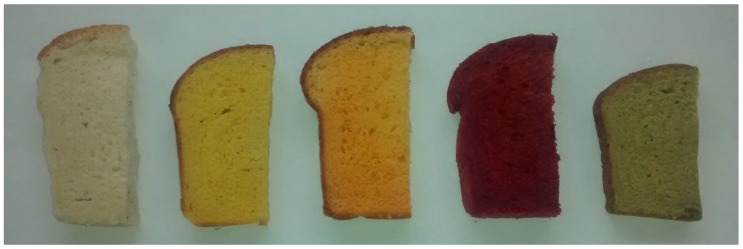
Physical appearance and size comparison of the baked breads. From left to right: Plain, Carrot, Tomato, Beetroot and Broccoli.

All five breads increased in hardness during storage. The hardness of carrot, tomato and beetroot breads were similar to that of the plain bread on the day of baking (Table 2). Broccoli bread was significantly harder compared to the plain bread (F(4,10) = 8.4; *p* < 0.05). This trend remained up to 2 days of storage. However by the 4th day of storage all four vegetable breads were similar in hardness to the control. The degree of cohesiveness showed a decreasing trend during storage and all breads showed a similar degree on the day of baking (*p* > 0.05). The cohesiveness of broccoli bread decreased significantly on days 1 and 2 whilst all the other vegetable breads remained comparable to the control. However, all the breads demonstrated similar values by 4 days of storage. Gumminess demonstrated an increasing trend during storage and all the breads showed statistically similar levels on the day of baking. Only broccoli bread showed a significantly different (higher) degree of gumminess at one day of storage compared to the control. However at days 2 and 4 all the vegetable breads demonstrated a similar degree of gumminess compared to the plain bread. The broccoli bread showed a poorer degree of leavening compared to all the other breads and was therefore smaller (Figure 4).

### 3.5. *In Vitro* Gastrointestinal Digestions

**Figure 5:**
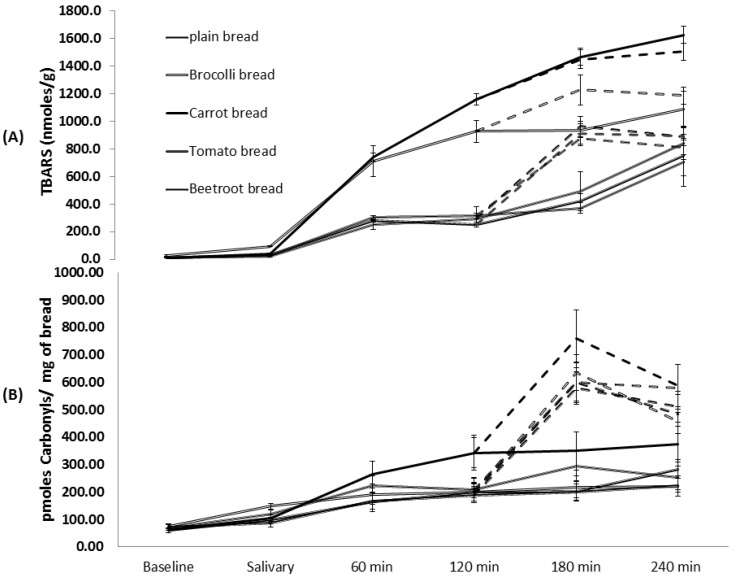
Temporal changes in TBARS (**A**) and protein carbonyls (**B**) production during gastrointestinal digestion of the breads. Solid and broken lines represent the salivary/gastric, and intestinal phases of digestion respectively. Values are means ± SE (*n* = 6).

The amount of TBARS generated during the oral phase of digestion was significant (F(4,25) = 17.86, *p* < 0.001) (Figure 5A). Post hoc analyses showed that broccoli bread generated a significantly higher amount of TBARS whilst all other vegetable breads produced similar amounts to the plain bread. Significant differences in TBARS production were observed during gastric digestion of the vegetables breads (F(4,25) = 38.05, *p* < 0.0001). Carrot and broccoli breads produced the highest and second highest levels of TBARS respectively. Post-hoc comparisons showed that TBARS content between these two breads were significantly different to each other, and both were significantly higher compared to plain, tomato and beetroot breads. The latter three breads demonstrated statistically similar TBARS contents. The intestinal phase showed a notable overall increase in the amount of TBARS produced in all the breads and the levels were significant (F(4,25) = 7.75, *p* < 0.001) (Figure 5A). Post hoc comparisons showed that carrot bread produced a significantly higher amount of TBARS compared to the plain, tomato and beetroot breads. Beetroot bread consistently showed attenuated TBARS levels at all three phases of digestion.

The vegetable breads produced statistically similar total amounts of PCs to the plain bread (Figure 5B). The total amount of PCs generated during the salivary, gastric and intestinal phases were not significantly different between the breads (F(4,25) = 1.78; *p* = 0.17, F(4,25) = 1.91; *p* = 0.14, and F(4,25) = 0.93; *p* = 0.46 respectively).

## 4. Discussion

The present study to our knowledge is the first comprehensive investigation that assesses the impact of adding vegetables to breads on shelf life, product quality and oxidative stability both during storage and digestion. The alimentary tract of humans can be an oxygen-rich environment and shows steep gradients both along the tract and within the lumen [4]. Furthermore, Gorelik *et al*. [21] showed that masticated bread dispersed in deoxygenated water resulted in the latter reaching oxygen saturation, which suggests that following the consumption of bread the chyme contains adequate levels of oxygen to induce a pro-oxidant environment. These reports underscore the importance of having adequate measures in place for protecting macronutrients from oxidation during digestion.

The study focused on breads that have fat added as an ingredient. Although traditional breads may not always contain fat most industrially processed variants contain up to 5% as it helps improve volume, texture and structure [22]. However, the addition of fat (predominantly unsaturated) also increases the oxidation potential of the product which could adversely affect sensory and storage properties. The inclusion of natural sources of antioxidants could be an effective strategy for improving oxidative stability of processed foods and would be a better and more consumer-friendly alternative to synthetic additives.

Since fruits and vegetables typically consist of up to 95% water using their dehydrated forms would enable the inclusion of greater loads in food formulations. The substitution of 10% of flour with dried vegetable powder in the present study resulted in a fresh vegetable equivalence of 300–900 g in an 800 g loaf which approximates to 30–90 g of fresh vegetables per 80 g portion of bread. Studies show that freeze drying produces a nutritionally superior product compared to hot air drying [23] as the absence of liquid water and the low processing temperatures minimise product damage and nutrient losses. Therefore, despite the higher associated costs freeze drying would be a nutritionally superior method for drying fruits and vegetables for use in food formulations. Freeze dried fruits and vegetables have significant but yet untapped industrial potential as an ingredient in processed foods as they could be used to improve nutritional quality, flavour, physico-chemical characteristics, as natural colourants, and in the formulation of speciality products such as low-gluten/gluten-free and vegan foods.

Fruit and vegetable consumption is generally associated with decreased morbidity from diet-related diseases. Although the specific mechanisms of action remain unclear it is thought to be in part due to the presence of antioxidants such as tocopherols, vitamin C, phenolics and carotenoids [24]. The present study also showed that the inclusion of vegetables significantly improved antioxidant potential. Interestingly beetroot showed the highest antioxidant potential in the FRAP assay and broccoli in the HORAC assay. Whilst these trends agree with previous findings [5] the divergent rankings may be due to differences in assay principles where the former measures reducing capacity and the latter quantifies the metal chelating radical prevention capacity. Therefore the results suggest that beetroot contains the greatest total antioxidant content whilst broccoli has the greatest potential to chelate metal ions and prevent the generation of hydroxyl radicals and resulting reactive oxygen species (ROS). This preventive antioxidant potential of broccoli may be due to the presence of glucosinolates which are suggested to be involved in metal chelation [25].

The inclusion of vegetables had significant and varying effects on oxidation products in the fresh breads. Whilst all the vegetable types reduced TBARS, carrot, beetroot and broccoli attenuated carbonyl formation. Overall, beetroot was the most effective in reducing both TBARS and protein carbonyls. This apparent macronutrient-protective effect of beetroot when included in processed foods agrees with previous observations [5,6,7] and is likely due to the presence of betalains, particularly betacyanin [26]. In agreement with previous observations [5] tomato did not attenuate carbonyls. The observation of a protein-protective effect of tomato may have been confounded by the production of endogenous carbonyls in the tomato tissue. Enzyme-mediated cleavage of endogenous unsaturated lipids has been shown to produce carbonyls in disrupted tomato tissue [27] which could have increased total carbonyl content in the fresh bread.

The study showed that adding vegetables into bread improved oxidative stability during storage. Compared to the plain bread the beetroot and broccoli breads demonstrated significantly longer induction times suggesting their addition prolonged shelf life. Beetroot had the greatest effect and this concurs with previous work showing that its addition to mayonnaise significantly improved induction times compared to other vegetables [7]. The same authors in another study using oil-in-water emulsions found that broccoli was the most efficient at increasing induction time [6]. Thus it appears that beetroot and broccoli can improve the shelf life of processed products. Interestingly, carrot and tomato breads showed shorter induction times than the control and these trends agree with previous studies showing negligible effects [6,7]. Similarly, Lee *et al*. [28] found that frying doughs containing carrot powder significantly reduced the oxidative stability of the frying oil. The pro-oxidant effects of carrot and tomato may be due to the presence of thermally degraded carotenoids (as a result of baking). Thermally degraded Lycopene and β-Carotene (heated to 90 °C) was shown to accelerate the oxidation of oil [29]. Conversely though, Duthie *et al*. [5] found that turkey burgers supplemented with tomato powder had the longest induction time compared to the control and other vegetable burgers. The core temperature of cooked burgers does not usually exceed 70 °C [30] and this milder thermal treatment may ensure the preservation of the antioxidant properties of carotenoids added to burgers. The antioxidant effects of ascorbic acid may also have been diminished in all the breads due to their thermal sensitivity and degradation at baking temperatures [31].

It is well known that Maillard reaction products that are formed during thermal processing have antioxidant effects. This has also been demonstrated in bread [32] and suggests that baking increases antioxidant levels and could further help protect against macronutrient oxidation. However, ascorbic acid and phenolics have shown to be consumed as reactants in the Maillard reaction and this may diminish their levels in the baked breads [33]. Yeast fermentation has been shown to improve antioxidant status by helping release bioactives from the food matrix and increasing free antioxidants and bioaccesability [34]. Thus the final antioxidant composition in baked products depends on complex interactions between the ingredients and processing conditions.

The absence of significant differences in hardness in fresh tomato, carrot and beetroot breads compared to the control is promising as it suggests that their addition had minimum effects on texture. The broccoli bread did not appear to prove as well as the other breads and the consequent higher density of the crumb was responsible for its greater initial hardness. The diminished leavening of the broccoli bread may have been due to antimicrobial compounds in broccoli hindering yeast activity. Yeasts, including *Saccharomyces cerevisiae* have been shown to be particularly sensitive to Sulphur compounds in Brassicas such as glucosinolates, Dimethyl Disulphide and Methanethiosulphonate [35]. Whilst the present study incorporated raw vegetables future work could determine if a better leavened bread could be produced by the use of brassica vegetables that have undergone pre-treatments such as heating to inactivate these compounds. The similar textural characteristics (hardness, cohesiveness and gumminess) of the vegetable breads to the plain bread after four days of storage indicates that vegetable inclusion did not adversely affect product storability and its physical characteristics associated with sensory appeal.

The present study for the first time investigated the antioxidant effects of vegetables incorporated into a processed food during gastro-intestinal digestion. Interestingly, carrot and broccoli appeared to increase lipid oxidation during oral, gastric and intestinal digestion phases, whilst the others had little effect compared to the plain bread. The reason for the greater TBARS formed during the digestion of carrot and broccoli breads is unclear and requires further study. These trends are not consistent with the levels of TBARS observed in the fresh breads which suggests that the digestive process is exacerbating TBARS generation in the case of some vegetables. Adding vegetables had no effects on protein carbonyl formation during gastro-intestinal digestion. Beetroot bread consistently showed low levels of oxidation products at all three digestive phases which indicates it is the most promising of the vegetables tested. All the breads showed higher TBARS and carbonyl levels during the intestinal phase compared to the gastric phase and this is likely to be due to pH differences. Previous work has shown that pH affects lipid oxidation and is greater at neutral compared to acidic conditions [36]. Although bile salts are an important functional component of lipid digestion our *in vitro* model did not contain it as preliminary experiments showed that it interfered with the TBA test, where the TBARS formed complexes with bile salts and produced underestimations. This previously unrecorded observation may suggest that bile plays a secondary but important role in reducing the absorption of TBARS from the chime, and justifies further investigation.

Sensory analysis is an essential aspect in reformulation studies and its absence is a limitation. The present experiments were conducted as a proof-of-concept study to determine the impact of vegetable addition on bread nutrition and oxidation status. As the results so far are encouraging the breads will be improved for sensory attributes at the next stage and a thorough sensory analysis carried out. Another limitation of the study was the absence of bread volume data. The measurement of bread volume would have provided a good indication of proving efficiency and structure of the vegetable breads.

In conclusion, adding vegetables to breads containing oil as an ingredient significantly improved nutritional and functional attributes of bread. Adding vegetables reduced lipid oxidation in bread. Carrot, beetroot and broccoli reduced protein oxidation and tomato increased it, whilst beetroot and broccoli improved shelf life. The addition of carrot, tomato and broccoli to bread showed equivocal effects on oxidative stability during gastrointestinal digestion and at times appeared to induce it. Beetroot consistently showed positive effects on macronutrient oxidation during storage and gastrointestinal digestion suggesting its addition to bread could be particularly beneficial.

## Figures and Tables

**Table 1:** Compositional information of the breads.

	Plain Bread	Broccoli Bread	Carrot bread	Tomato Bread	Beetroot Bread
Moisture	37.6	37.3	36.9	37.5	36.7
Protein	17.1	13.9	11.4	12.0	11.8
Total Carbohydrates	68.2	59.3	58.8	60.3	60.2
Fat	11.3	11.9	12.2	11.8	11.4
Ash	2.8	3.3	3.1	3.4	3.2
NSP	4.3	7.1	6.7	6.5	6.4
α- and γ-Tocopherol	39.9	93.7	22.8	70.7	30.1
α-Carotene	-	-	31.0	-	-
β- Carotene	0.2	23.3	119.0	32.3	0.2
β-Cryptoxanthin	-	-	0.5	-	-
Lutein/Zeaxanthin	2.9	28.3	3.6	6.0	3.4
Lycopene	-	-	4.6	97.5	-

Protein was measured as N and converted using a factor of 6.25; Tocopherols and Carotenoids are in µg/g of dry matter, Proximate values are g per 100 g of dry matter; NSP = Non-Starch Polysaccharides (Rhamnose, Fucose, Arabinose, Xylose, Mannose, Galactose, Uronic acid); Blank cells indicate no detectable levels.

**Table 2:** Changes in the texture profile of bread during storage.

	Hardness (g)	Cohesiveness	Gumminess (g)
Day 0			
Plain	279.3 ± 16.7 ^a^	0.58 ± 0.2 ^a^	192.0 ± 31.2 ^a^
Carrot	488.7 ± 24.9 ^a^	0.43 ± 0.1 ^a^	257.7 ± 41.6 ^a^
Tomato	392.0 ± 38.1 ^a^	0.44 ± 0.1 ^a^	237.0 ± 44.5 ^a^
Beetroot	696.7 ± 73.6 ^ab^	0.47 ± 0.0 ^a^	335.7 ± 49.0 ^a^
Broccoli	1304.0 ± 434.3 ^b^	0.42 ± 0.1 ^a^	424.7 ± 236.0 ^a^
Day 1			
Plain	409.0 ± 8.5 ^a^	0.41 ± 0.0 ^a^	193.3 ± 28.5 ^a^
Carrot	733.7 ± 35.5 ^ab^	0.41 ± 0.0 ^a^	360.3 ± 82.1 ^ab^
Tomato	682.3 ± 59.8 ^ab^	0.34 ± 0.0 ^ab^	260.7 ± 54.9 ^a^
Beetroot	785.7 ± 40.5 ^ab^	0.37 ± 0.0 ^ab^	344.3 ± 5.6 ^ab^
Broccoli	1220.7 ± 469.3 ^b^	0.32 ± 0.0 ^b^	573.0 ± 110.2 ^b^
Day 2			
Plain	616.0 ± 92.0 ^a^	0.37 ± 0.0 ^a^	315.7 ± 105.5 ^a^
Carrot	775.7 ± 113.0 ^ab^	0.36 ± 0.0 ^ab^	348.7 ± 66.3 ^a^
Tomato	787.3 ± 111.3 ^ab^	0.31 ± 0.0 ^ab^	288.7 ± 15.3 ^a^
Beetroot	1082.7 ± 53.0 ^ab^	0.32 ± 0.0 ^ab^	384.7 ± 2.9 ^a^
Broccoli	1432.0 ± 367.6 ^b^	0.28 ± 0.0 ^b^	560.0 ± 119.4 ^a^
Day 4			
Plain	915.0 ± 133.0 ^a^	0.34 ± 0.0 ^a^	343.7 ± 62.6 ^a^
Carrot	991 ± 149.7 ^a^	0.31 ± 0.0 ^a^	412.3 ± 83.6 ^a^
Tomato	965.0 ± 233.3 ^a^	0.28 ± 0.0 ^a^	276.3 ± 71.4 ^a^
Beetroot	1310.7 ± 33.4 ^a^	0.29 ± 0.0 ^a^	414.7 ± 59.5 ^a^
Broccoli	1649.0 ± 544.4 ^a^	0.31 ± 0.0 ^a^	533.3 ± 240.3 ^a^

Values are means ± standard deviations (*n* = 4); Values with different superscripts within a column for each day are significantly different (*p* < 0.05); Cohesiveness is unit-less.
